# Barriers to self-management engagement among community-dwelling older adults with mild cognitive impairment: a qualitative study

**DOI:** 10.3389/fpsyt.2024.1446344

**Published:** 2025-01-07

**Authors:** Jingxian Sun, Yayi Zhao, Aihong Wang

**Affiliations:** School of Nursing, Nanjing University of Chinese Medicine, Nanjing, China

**Keywords:** mild cognitive impairment, older adult, community-dwelling, self-management, barriers, qualitative study

## Abstract

**Introduction:**

Self-management is crucial for individuals with mild cognitive impairment (MCI) to enhance cognitive health and mitigate the potential risk of dementia. However, maintaining consistent engagement in self-management strategies seems a challenge for older adults with MCI. This study sought to gain insights into the barriers to self-management engagement among community-dwelling older adults with MCI.

**Methods:**

This qualitative study used a phenomenological approach. Purposive sampling was used to recruit a diverse group of participants with MCI, aged 60 and above, residing in the provinces of Hubei, Jiangsu, Henan, and Anhui in China. Semi-structured interviews were conducted between January and May 2023, allowing for an in-depth exploration of participants’ experiences and perceptions. The interviews were audio-recorded, transcribed verbatim, and subjected to thematic analysis to capture the essence of the participants’ narratives.

**Results:**

Twenty-six participants participated in the formal semi-structured interviews. The following themes and subthemes were identified: “inappropriate perceptions of MCI” (“low perception of risk from MCI” and “supposition of little personal control over MCI”), “elder self-neglect”, and “lack of available information or support services about MCI”.

**Discussion:**

This study highlights significant barriers to self-management engagement among community-dwelling older adults with MCI, emphasizing the importance of developing tailored interventions that address misconceptions and self-neglect and enhance the availability of information and support services. These findings suggest avenues for policymakers and healthcare providers to foster more effective self-management practices in this population, promoting cognitive health and slowing potential progression to dementia.

## 1 Introduction

Mild cognitive impairment (MCI) is the preclinical, transitional stage between healthy aging and dementia (1, 2). The global prevalence of MCI is reported to account for >15% in community-dwelling adults aged ≥50 years and increase with age (3). As MCI is prone to progress to dementia (1, 4, 5), the large MCI population has become a public health concern worldwide (6, 7). Measures to halt or slow potential disease progression are crucial to forestalling further cognitive decline and reducing the prevalence of dementia (1, 2, 8). Because of the lack of recommended pharmacological interventions, a consensus has been reached that management strategies, including physical exercise, cognitive training, addressing modifiable risk factors, and regular monitoring of cognitive status, should be adopted by people with MCI to promote cognitive health and attenuate potential dementia onset (1, 9, 10).

As MCI is a chronic condition, the level of compliance with participation in cognitive health management strategies, that is, self-management engagement, is the primary factor affecting the management effect and disease outcome (11, 12). A higher level of self-management has been shown to be associated with greater improvement in cognitive performance among older adults with MCI, whereas poor self-management can undermine management effectiveness (13, 14).

However, sustainable participation in various such strategies seems a challenge for older adults with MCI. A 12-month cluster randomized controlled trial (RCT) was conducted to examine whether four structured lifestyle activity interventions might optimize cognition among older adults with MCI; the adherence rate of integrated cognitive–physical exercise among participants was only 65% (13). A 4-month home-based strength–balance exercise program for individuals with MCI and early dementia showed that only 60% of participants met adherence expectations, while the remaining participants demonstrated low adherence (15). Moreover, after finishing interventional programs, older adults with MCI might find it more difficult to continue self-management without supervision from professionals. Notably, in one study, only one fourth of older adults with MCI maintained compliance with management strategies after completing an exercise program (14).

One of the general principles of chronic disease management is to enhance individual empowerment and strengthen individuals’ disease self-management behaviors (16, 17). Thus, there is a critical need to explore the specific barriers that community-dwelling older adults with MCI encounter in their self-management practices. This qualitative study aimed to gain insights into the barriers to self-management engagement from the perspective of community-dwelling older adults with MCI. The findings of this study are expected to contribute to the development of targeted interventions that can address the identified barriers, thereby promoting self-management and cognitive health and mitigating the potential risk of dementia among older adults with MCI.

## 2 Methods

### 2.1 Design and participants

This was a qualitative study that used a phenomenological approach. We recruited participants from communities in Hubei, Jiangsu, Henan, and Anhui Provinces in China. Inclusion criteria of participants in this study were as follows: (1) age ≥60 years, (2) diagnosed with MCI, (3) community-dwelling, (4) participating voluntarily, and (5) able to provide informed consent. In this study, we used the diagnostic criteria for MCI established by Petersen (18): (1) cognitive complaint by the patient, the patient’s informant, or the physician; (2) cognitive decline that is not normal for age; (3) essentially normal functional activities; and (4) no dementia.

Potential participants were alerted to the study through flyers posted on community billboards or through direct physician referrals at local community health service centers. The researchers approached potential participants face to face and explained the details of the study to invite their participation. Diagnoses of MCI were verified by clinicians with expertise in cognitive disorders through standardized assessments at tertiary, top-tier hospitals. Individuals with impaired communication were excluded. Those who were willing to participate signed their written informed consent and provided demographic and clinical data.

### 2.2 Data collection

The research team consisted of three researchers from a medical university in Central China. JS has an MSN degree, whereas YZ and AW have a PhD degree. All were familiar with the topic and had experience in interviewing people with MCI. To ensure the quality of data collected, all researchers received intensive training in qualitative research methodology before the study. There were no relationships between the researchers and the participants before the study commenced. At the time of recruitment, participants were informed about the details of the study and the background of the researchers.

A semi-structured interview guide was developed based on a literature review, including the following questions with probes. (a) Could you describe your understanding of self-management in relation to your cognitive health? (b) Reflecting on your experiences, what does engaging in self-management activities mean to you personally? (c) What challenges or barriers have you encountered when attempting to consistently engage in self-management strategies? How do these challenges or barriers affect your motivation or ability to manage your cognitive health? (d) Were there instances when you felt particularly discouraged from pursuing self-management strategies? Can you describe those instances? (e) What kind of support do you feel you need to effectively engage in self-management? Are there resources that you find lacking or that you wish were more accessible to you? (f) Is there anything else about your experiences with self-management and cognitive health that you think is important to share? Pilot interviews were conducted with two participants to identify and address any potential problems. The data from pilot interviews were not included in the analysis.

Participants who had provided signed written consent were contacted in advance to schedule the interview time and place according to their convenience and willingness. We conducted one-on-one, face-to-face interviews either in a quiet room of a community health service center or in the participant’s home without disturbance. Each interview was audio-recorded with the participant’s explicit consent. During the interviews, questions from the interview guide were used, and participants were also asked for more information in spontaneous follow-up questions to expand on particular points that came up in the interview. Meanwhile, we took field notes on participants’ body language and emotions. Interviews continued until data saturation was achieved (*i.e.*, no new data emerged) (19).

### 2.3 Data analysis

JS transcribed the audio recordings verbatim immediately after each interview. YZ checked the transcripts for accuracy. Thematic analysis by Braun and Clarke was employed for data analysis (20). The analytical process included the following steps: (1) familiarize oneself with the data, (2) generate initial codes, (3) search for themes, (4) review themes, (5) define and name themes, and (6) produce the report. We used NVivo software version 12 (Lumivero, Denver, CO, USA) to facilitate data management and analysis. Two researchers analyzed the transcribed data independently after each interview. Then, we conducted group discussions to compare the findings until a consensus was reached. This process of independent analysis followed by consensus work continued until data saturation was achieved.

### 2.4 Rigor

Trustworthiness was evaluated using Lincoln and Guba’s criteria (21), including credibility, transferability, dependability, and confirmability. All researchers had experience in interviewing people with MCI and were trained in qualitative research methodology prior to the study. The semi-structured interview guide was developed on the basis of a comprehensive literature review. Two researchers analyzed the data independently, and group meetings were conducted to reach consensus on the findings. The above-described methods enhanced the credibility of the study. To ensure transferability, we formulated a thick description of the context, study design, participants, and data collection and analysis. To ensure dependability and confirmability, we maintained an audit trail that included a complete set of notes to facilitate tracking the steps of the study. Participants’ interview statements were directly quoted to support the findings. Finally, we kept a reflexivity diary to bracket our personal views and preconceptions and ensure that the findings were grounded in the data.

### 2.5 Ethical considerations

This study was approved by the ethical review board of the researchers’ university. All participants voluntarily participated in the study and provided written informed consent. They were informed that they could withdraw from the study at any time without further explanation and that their personal information would be encrypted.

## 3 Results

Data saturation was attained at the 24th interview. We then recruited two additional participants to confirm data saturation. A total of 26 participants participated in formal interviews from January to May 2023, achieving theoretical saturation. Interviews lasted 42–67 min. Demographic and clinical characteristics of the participants are presented in **Table 1**.

**Table 1:** Demographic and clinical characteristics of interviewees (N = 26).

Characteristics	Count	%	Score
Gender			
Male	12	46.15%	
Female	14	53.85%	
Age (years)			
60–69	13	50.00%	
70–79	11	42.31%	
≥80	2	7.69%	
Education			
Elementary school or below	15	57.69%	
Junior high school	2	7.69%	
High school or above	9	34.62%	
Marital status			
Married	24	92.31%	
Widowed/divorced	2	7.69%	
Monthly income ($)			
<300	10	38.46%	
300–600	8	30.77%	
>600	8	30.77%	
Employment status			
Employed	9	34.62%	
Retired	17	65.38%	
Residence			
Rural area	15	57.69%	
Urban area	11	42.31%	
Comorbid condition			
Yes	18	69.4%	
No	8	30.6%	
MoCA			20.46 ± 1.334^a^

MoCA, Montreal Cognitive Assessment.

a Average score.

To explore the barriers to self-management engagement among community-dwelling older adults with MCI, the following themes and subthemes were identified: “inappropriate perceptions of MCI” (“low perception of risk from MCI” and “supposition of little personal control over MCI”), “elder self-neglect”, and “lack of available information or support services about MCI”.

**Theme 1: Inappropriate perceptions of MCI**

**Subtheme A: Low perception of risk from MCI**

Because the symptoms of MCI are relatively insidious, the participants thought MCI did not, and would not, greatly affect their daily lives.

*Well, it’s only memory loss. It’s not a big deal for old people, you know. It doesn’t bother me because it doesn’t make much difference to ordinary life.* (ID 21)

*I think my condition is OK. Everything goes normally. I don’t think I need to worry about it.* (ID 05)

Meanwhile, as participants became increasingly cognizant of the overall decline in physical function that came with age, they accepted the reality and reduced demands on themselves, focusing more on basic activities of daily living and less on advanced abilities such as cognitive function. Therefore, although participants might have experienced some symptoms of MCI, they did not perceive them as concerning. They expressed the opinion that their conditions could still be managed, since they did not need to engage in complicated work at their ages.

*Well, you see, I’m already 74 years old. As long as I’m fed and warm, that’s enough. As for forgetfulness, it doesn’t affect my life obviously. What could I ask for more at this age?* (ID 24)

*My life has not been affected, and I do not feel any sense of crisis. I get up in the morning, eat breakfast, send my grandson to school, and pick him up in the afternoon. I only need to know which kindergarten my grandson is in, when to send him to school, and when to pick him up. That’s all. That’s enough.* (ID 07)

In addition, due to their lack of factual knowledge on the potential course or prognosis of MCI, participants generally perceived little future risk from their conditions.

*Dementia? I don’t think MCI has anything to do with dementia. It’s not as serious as dementia, you know. Old men always have problems, this or that, you know. Nothing to worry about.* (ID 03)

**Subtheme B: Supposition of little personal control over MCI**

Almost all participants perceived MCI as an unavoidable consequence of aging. They believed that symptoms of MCI such as memory loss would inevitably appear with age due to the nature of aging.

*With age, MCI is inevitable, you know? It’s inevitable, definitely. When you get old, all the organs and nerves that belong to your body become weakened, you know, no one could escape.* (ID 22)

*At this age, you know, all functions are getting worse. I am an old man now. I am no longer a young man. How could it be the same? Just like a machine, after a long time of use, there would be rust and loose screws and so on. That is normal, you know, that is unavoidable. So does MCI.* (ID 02)

The majority of participants did not suppose that there were current effective treatments or interventions for MCI.

*I don’t think MCI could be treated. It’s aging, you know. No one could stop it, no one. There is nothing we could do. We have to let it run its course.* (ID 04)

*The point is you’re old, and nothing could change it. Take deafness, for example. If you are young, it might be cured. But if it is age-related deafness, how could it be cured? You must always remember that you are no longer young. The eye problems of young people usually can be cured. But an old man’s presbyopia could never be cured, [and] neither [could] MCI. That’s it. You have to accept it.* (ID 23)

**Theme 2: Elder self-neglect**

Participants frequently conveyed sentiments of diminished self-worth in their later years.

*Now that I’ve reached this age, what use am I? When people grow old, they become useless.* (ID 26)

*I’m getting old, and I don’t know when I’ll pass away. It’s meaningless. What is there to think about?* (ID 05)

Elder self-neglect was evident in participants’ apathy toward their health conditions, which significantly eroded their motivation and confidence in managing their cognitive health.

*Sigh, at my age, do I still need to improve my cognitive function? What’s the use even if I do? It’s enough just to be alive.* (ID 12)

*Cognitive functions, cognitive impairments, I don’t care about any of that. At this age, what is there left to fuss about? Just eat, drink, and whenever death comes, so be it.* (ID 25)

**Theme 3: Little available information or support services about MCI**

Most of the participants lacked factual knowledge about MCI to some extent.

*I don’t know much about MCI. It was the first time I heard about it, when I was diagnosed.* (ID 02)

*To be honest, I don’t know what MCI is, exactly. What is cognitive function? I could not get it, either. I don’t know exactly what is going on and how to cope with it.* (ID 08)

Other participants expressed similar statements many times. Participants reported a lack of access to knowledge about MCI, of publicity and education about MCI in the community, and of assessments during regular health check-ups or other medical visits.

*There seems to be no way to know about MCI, you know. No one talks about it in daily life. No publicity and education about it in the community. I had never heard about it until I was diagnosed, [and] neither [had] my family. Nobody ever told us about it or even mentioned it, even the doctors.* (ID 15)

*There’s no cognitive assessment in routine health check-ups. The check-ups are generally conducted in the community health service center, including B[-scan] ultrasound, blood test, urine test, and blood pressure measurement. That’s all. [A] cognition test or memory test was not included.* (ID 17)

These statements were representative of most participants’ experiences. In addition, the majority of participants indicated that their family members did not pay much attention to their condition nor talk about it with them.

*My family did not pay attention to it [MCI]. We never talked about it. They think my cognition is pretty normal.* (ID 04)

*They* [family members] *are very busy with their work. They don’t have time for it* [MCI]. *They think it is not a big deal.* (ID 08)

Even though a small minority of family members noticed the participants’ cognitive decline, they did not take corresponding measures due to their lack of relevant knowledge.

*My daughter thinks my memory has declined apparently. She is worried about the possibility of dementia in the long run. But she doesn’t know what to do.* (ID 21)

## 4 Discussion

The findings from this qualitative study highlight significant barriers to self-management engagement in community-dwelling older adults with MCI. A recurring theme in participant narratives was the presence of inappropriate perceptions of MCI, including a low perception of risk and a supposition of little personal control over the condition. There appears to be multiple factors influencing a person’s perceptions about MCI, such as expectations of normal aging, personal experience with dementia, social support networks and concurrent health problems, leading to varying subjective interpretations of the meaning of MCI (22, 23). Some studies indicated that (24–27), as ‘life goes on as normal’, individuals with MCI perceived little seriousness of the potential consequences of MCI and the majority of them had no recollection of any discussion around the likelihood of progression, which are consistent with our findings. Nevertheless, other study also reported the experiences of worrying about dementia and further cognitive deteriorations among individuals with MCI (28). There are also differences in the perceptions regarding the controllability of their condition among older adults with MCI. One study indicated that these individuals believe MCI is manageable (29), while another study reported that they consider MCI to be uncontrollable (30), similarly to our findings. These findings resonated with the health belief model, which posits that individuals are less likely to engage in health-promoting behaviors if they do not perceive a threat or if they feel that their actions will not effectively prevent or ameliorate the condition (31, 32). Accurate patient understanding of the illness would allow appropriate coping behaviors (33–35). Therefore, developing interventions against perceptions that act as barriers to optimal coping skills and health outcomes is urgently needed. Studies suggested that psychoeducation could change misconceptions or address knowledge gaps in ways that improve illness perceptions and health outcomes (36–40). Therefore, it could be used to clarify potential confusion or knowledge gaps around MCI on the part of patients and their families’. Further efforts to tailor patient education to patient baseline perceptions and cognitive levels are recommended in clinical practice.

The identification of elder self-neglect as a barrier adds a layer of complexity to the self-management of MCI. Elder self-neglect, which commonly refers to refusal or failure to provide oneself with adequate water, food, shelter, clothing, medication, or safety precautions, is a global public health and human rights issue that threatens older people’s health and safety (41). Evidence has shown that a decline in executive function was associated with a risk of elder self-neglect and that a decline in global cognitive function was associated with a risk of greater self-neglect severity (42, 43). Thus, older adults with MCI are prone to self-neglect. Self-neglect is associated with adverse outcomes concerning older people’s physical, cognitive, and psychological well-being (44–47). It has been reported that medication non-adherence is a very prevalent problem among self-neglecting older adults, which could undermine self-management efforts and exacerbate the existing medical conditions (41, 48, 49). Similar to our study, Zhang et al. conducted a qualitative study to explore perceived challenges of self-management in older people with hypertension, and the results reported that participants demonstrated elder self-neglect as a challenge concerning hypertension self-management (50). The reasons behind self-neglect can be multifaceted, often rooted in psychological, social, and physiological factors (51). As a vulnerable population at risk of self-neglect, individuals with MCI require multifactorial interventions that not only address their medical needs but also support their emotional and social well-being.

The lack of accessible information and support services may significantly contribute to misconceptions about MCI and act as a barrier to self-management engagement among individuals with the condition, as indicated by our findings. A nationwide online survey among the Chinese public indicated that only 17% of the respondents chose to see a doctor for perceived memory or cognitive problems (52). According to another national survey in China (7), 6,926 (97.2%) of 7,125 individuals with MCI had never seen a doctor regarding their condition nor taken any measures before the screening. A similar situation also exists in other countries worldwide. A survey commissioned by the Alzheimer’s Association reported a reluctance among Americans to see their doctor after noticing MCI symptoms (53). These findings highlight the necessity for accessible information and support services regarding MCI, not only for individuals affected by this condition but also for the general public. Various campaigns, from community-based education programs to national initiatives, offer important avenues to raising awareness of MCI, locally and nationally (52, 54). Many factors must be taken into consideration to tailor the campaign strategy to the target audience, including that audience’s age, gender, socioeconomic status, culture, and geographic location. An appeal has been launched to all stakeholders, including policymakers, health and advocacy organizations, health professionals, and patient groups, to lend their support to these campaigns (55).

We also found that family members did not help facilitate positive coping for older adults with MCI. Close family members are typically the first to notice cognitive changes, yet many are reticent to discuss the topic. Because illness perceptions are interdependent within family units (27), MCI-related education and support should also engage patients’ close relatives. It is imperative to launch campaigns aimed at encouraging families to discuss cognitive concerns with each other and provide support for active management of cognitive health, as recommended by the Alzheimer’s Association (54).

As reported in this study, cognitive assessments are not currently included in regular health check-ups or routine medical visits in China. An international call has gone out for early detection of cognitive impairment as a patient’s right (56). Casefinding by health professionals is considered an important step toward cognitive-health enhancement for aging populations. Therefore, the establishment of a national surveillance network that performs regular cognitive evaluations of people age ≥60 years to monitor their changes in cognition and identify early cognitive impairment is strongly recommended (7). Meanwhile, practicable management strategies and support services should also be provided to patients with MCI. Correspondingly, the medical-insurance system in China should be reformed to reimburse patients for cognitive assessments and care plan services, as is done in some other countries (57). As reported by the National Health Commission of China (58), the Chinese government has launched an initiative to include management of cognitive impairment among older adults as an important part of China’s public-health system. Pilot projects for prevention of and intervention into dementia have been organized in 15 provinces to improve the cognitive health of the elderly. The government has also emphasized that early recognition and guidance of MCI in the elderly would be further strengthened in the future.

It is also pertinent to consider the cultural context within which this study was conducted, as cultural norms and beliefs regarding aging and cognitive decline may influence perceptions and engagement in self-management (59, 60). Thus, when applying the results of this study to other contexts, it is important to consider that the cultural backgrounds of different locations may affect the applicability of the findings. Simultaneously, in developing intervention strategies aimed at enhancing self-management behaviors among older adults with MCI, it is crucial to consider the local cultural context to ensure that the interventions respect and integrate local customs and beliefs and maximize their effectiveness.

### 4.1 Strengths and limitations

This study had several strengths. First, this study used a qualitative phenomenological approach, which was well-suited for exploring the lived experiences and perceptions of community-dwelling older adults with MCI, allowing for an in-depth understanding of the barriers to self-management engagement from the participants’ perspectives. Second, the sample included a group of participants recruited from multiple provinces in China, and the participants varied in demographic characteristics such as ages, genders, regions, careers, or incomes. This diversity contributed to the richness of the data and enhanced the generalizability of the findings.

The limitations of the study must also be considered when interpreting our data. First, although the study included participants from several provinces, the findings may not be generalizable to the entire MCI population around the country or internationally because of the small sample size. Second, the study focused on a specific cultural and regional context, which may limit the applicability of the findings to other cultural settings. The barriers identified may be influenced by specific cultural attitudes and healthcare systems, which may not be universally applicable.

## 5 Conclusion

The study highlighted significant barriers to self-management engagement among community-dwelling older adults with MCI in China. The findings reveal that misperceptions about MCI, including the underestimation of its risks and a belief in limited personal control over the condition, contribute to disengagement in self-management practices. Additionally, elder self-neglect and the scarcity of information or support services further hinder the ability of these individuals to effectively manage their condition. Addressing these barriers by enhancing awareness and understanding of MCI, alongside improving access to resources and support services, is essential for promoting active and effective self-management among older adults with MCI. Future interventions should focus on educational programs tailored to this demographic to clarify the nature and risks of MCI and empower individuals with the knowledge and support necessary for effective self-management.

## Data Availability

The original contributions presented in the study are included in the article/supplementary material. Further inquiries can be directed to the corresponding author.
